# Acute Abdominal Pathology in Pregnant Women

**DOI:** 10.5334/jbr-btr.1035

**Published:** 2016-02-15

**Authors:** Filip Claus

**Affiliations:** 1 OLV Hospital Aalst, BE

Imaging pregnant patients comes with several challenges, in terms of the type of pathology (maternal, fetal or placental), choosing the optimal imaging modality and minimizing the radiation risk. Radiation exposure information is sometimes limited and confusing, for both referring physicians and patients, and may lead to uninformed decisions on the appropriateness and risks of the exam. Overestimating a negligible risk following a low radiation dose can induce excessive and unnecessary anxiety among patients or delay diagnosis and treatment. Underestimation of the risk can result in fetal non-carcinogenic (teratogenic) effects and both fetal and maternal carcinogenic effects.

Fetal radiation doses, above 100 and 150 mGy respectively, bear a potential and high non-carcinogenic risk. The reported lifetime attributable risk of cancer incidence for fetuses exposed to ionizing radiation (X-ray and computed tomography) ranges between 2.5 and 5% per 100 mSv. Magnetic resonance (MR) imaging (1.5 and 3.0 Tesla) can be performed in all trimesters if deemed clinically necessary (and no delay until completion is possible) and ideally as an adjunct to initial ultrasound evaluation. Although no teratogenic adverse effects are reported for iodinated and gadolinium based contrast agents, informed consent is recommended when their use is considered clinically necessary.

A typical example of the use of abdominal computed tomography in pregnant patients is a severe/high-velocity trauma.

Placental pathology is often less known by radiologists. Following an inconclusive sonographic examination, MR is the imaging modality of choice for the placenta. A minimal MR checklist should examine the location and morphology of the placenta, document placental and umbilical cord anomalies and look for placental invasion. The MR key signs of placental invasion are an abnormal uterine bulging, heterogeneity of the signal intensity within the placenta, and the presence of dark intraplacental bands on T2-images. One should note that radiological techniques often fail to accurately determine the extent of placental invasion.

Fetopelvic disproportion, particularly in primigravida, typically presents late in pregnancy. Maternal pelvimetry might be a helpful adjunct to clinical parameters and examination. X-ray pelvimetry should be avoided, because of the relatively low accuracy of the modality, the high interobserver variability and the use of ionizing radiation. MR is the modality of choice to measure the pelvic diameters and if not available, low-dose computed tomography might be an alternative. When using pelvimetry reference values, one should pay attention to the number of participants included in the study, the imaging modality used in the study and the reason for pelvimetric imaging of the patient group (e.g. spontaneous deliveries, suspected fetal-pelvic disproportion, all referred patients, etc.).

## Competing Interests

The author declares that they have no competing interests.

